# Postnatal depression in Southern Brazil: prevalence and its demographic and socioeconomic determinants

**DOI:** 10.1186/1471-244X-8-1

**Published:** 2008-01-03

**Authors:** Leila Tannous, Luciana P Gigante, Sandra C Fuchs, Ellis DA Busnello

**Affiliations:** 1 Medical Science, School of Medicine – Universidade Federal do Rio Grande do Sul. Psychiatrist of Secretaria da Saúde, Porto Alegre City Hall and Hospital Psiquiátrico São Pedro, Brazil; 2 School of Medicine and Postgraduate Program in Public Health – Universidade Luterana do Brasil, Brazil; 3 Department of Social Medicine and Postgraduate Program in Medical Sciences: School of Medicine – Universidade Federal do Rio Grande do Sul, Brazil; 4 Psychiatry Department of Psychiatric and Forensic Medicine: School of Medicine – Universidade Federal do Rio Grande do Sul, Brazil

## Abstract

**Background:**

Studies investigating the prevalence of postnatal depression (PND) show rates ranging from 5% to 36.7%. The investigation of age, race, educational levels, religion and income as risk factors for PND has yielded conflicting results. The aim of this study is to investigate the prevalence of PND in women residing in Southern Brazil and the associated risk factors.

**Methods:**

This is population-based cross-sectional study of women residing in Porto Alegre who delivered in June 2001. A sample of 271 participants were selected from the Record of Living Newborn Infants of the State Health Department (the official Brazilian database and stores the name and address of all women who give birth to living newborn infants) using a process based on pseudo-random numbers which choose a random sample from 2.000 records. Once the addresses were identified, the women were visited at their place of residence (home, hotel, boarding house and prison), with the interviews taking place between the 6^th ^and the 8^th ^week after delivery.

The association between the risk factors and PND was investigated through bivariate analysis using Pearson's chi-square test. Student's t-test was used to analyze the continuous variables. To identify independent risk factors, multivariate analysis was performed using hierarchical levels with a predefined model that took into account the time relationship between PND and the risk factors. Cox's regression was used to calculate the prevalence ratios.

**Results:**

The PND prevalence rate found was 20.7% (CI 95% 15.7 – 25.7). After adjusting for confounding variables, per capita income was found to have a significant association with PND.

**Conclusion:**

The prevalence of PND is higher than the figures found in most developed countries and similar to the figures found in developing countries. Differences in PND by regions or countries can be partially explained by the effect of income on the mediation of risk factors. In low income populations, women should be routinely evaluated for postnatal depression, and those with no partner or spouse are likely to require further care from health services and should be given the benefit of mental health prevention programs.

## Backgroud

Postnatal depression (PND) is a condition characterized by a persistent experience of sadness or a diminished ability to experience pleasure, irritability, feelings of low self-esteem and manifest anxiety [1], a tendency to brood over the infant's health and well-being [2], fatigue, as well as alterations [1] in sleep patterns and appetite. The onset of PND is usually [3] seen after the fourth week after birth but it may appear up to the end of the first year after birth. A difficulty in performing household chores and mood disturbances are some of the symptoms that often go unreported [1,4] to other people. According to DSM-IV [5]., postnatal depression does not differ from other depressive conditions except for its onset period – within the first 4 weeks postpartum.

Most women suffering from PND do not receive any form of treatment [1,6] and may remain depressed for up to a year after delivery [1,7], a situation which may seriously compromise the development of the mother-infant bond [8], cause delays in the cognitive and emotional development of the newborn infant [9] and result in abuse and negligence [10] in the child's care. In addition, this may also affect the relationship with the partner [11] and is a risk factor for new episodes of depression [12] for a period of five years thereafter.

PND prevalence rates range from 5% in Aarhus, Denmark [13,14] and Jerusalem, Israel [15,14] to 36.7% in Santiago [16], Chile. In Brazil, studies using the Edinburgh Postnatal Depression Scale (EPDS) detected prevalence rates of 12% in Rio de Janeiro [17] and 13.4% in [18] Brasilia. In Southern Brazil, a study [19] using the Hamilton scale found 19.1% of mothers suffering from depression.

Studies have identified characteristics such as age [15,20] race [17], educational level [21,22], and religion [15] as risk factors; however, some results are inconsistent.

The identification of PND-affected women and the treatment provided to them are a reality in industrialized countries. In developing countries, the identification of afflicted populations and groups potentially requiring attention are key factors for the organization and adequacy of health services and the planning of health programs.

This study investigated the prevalence of PND in a representative population-based sample of women residing in Porto Alegre and its associated demographic, socioeconomic and religious orientation-related risk factors.

## Methods

### Study design

A population-based cross-sectional study of women residing in Porto Alegre who gave birth to living newborn infants between the 5^th ^and the 18^th ^of June, 2001. Simple random sampling was used to select participants from the record of *Living Newborn Infants*, a Database of the State Health Department. This is the official Brazilian database and stores the name and address of all women who give birth to living newborn infants using the data supplied by the hospital where the baby was delivered or the birth register office in case of home delivery.

According to data from the Record of Living Newborn Infants of the Porto Alegre Health Department, every child born to a mother residing in Porto Alegre and alive up to the first minute has their record entered.

Porto Alegre, state capital of Rio Grande do Sul, is located in Southern Brazil. It has [23] a population of 1,416,735, area of 496.8 km^2 ^and an annual per capita income of U$ 4840.91. Its economy is based on the industry, commerce and services. In Brazil, health is a state's obligation and its access is universal. As in the rest of the country health, education and safety have both public and private services. By and large, 2/3 of the population uses the government's services.

In Porto Alegre, the public health services are distributed into 8 sanitary districts. Each sanitary district encompasses a number of neighborhoods, and there are primary health centers in every neighborhood in the city. These sanitary centers develop national-level programs, the ones providing attention for pregnant women and first infancy being considered priority. There are follow-up programs for women with high-risk pregnancies as well.

### Sample size calculation

Historical data shows that, on average, 2,000 women give birth during the month of June. The sample size was based on a 12% prevalence of PND [24] and a precision level of ± 4% around the prevalence rate, with a confidence interval of 95%.

The sample size was also calculated to investigate the risk factors for PND. The interruption of breastfeeding was chosen as a risk factor of interest given the fact that it is observed in 60% of depressed mothers, a figure estimated on a population-based study [25] carried out in a nearby city showing 50% of ablactation at 2 months. We identified that 233 women should be investigated so as to detect a prevalence ratio of 3.0 with a confidence interval of 95% and power of 80%, assuming that 7% of breastfeeding women would suffer from PND. An additional 67 women were added to the sample size in order to maintain the power of the study in the multivariate analysis should there be any dropouts or refusals. Calculations were performed using software Epi-info 6.04.

The inclusion of breastfeeding or interruption thereof as a PND-associated risk factor for purposes of sample size calculation was based on evidence indicated by the literature: breastfeeding or interruption thereof is counted among those risk factors [20-22,24] most frequently studied. Results regarding this association are presented in an article by the same authors, submitted for publication.

The study was submitted to the Research Ethics Committee of the Porto Alegre City Hall Health Department and to the Research Ethics Committee of the *Hospital de Clínicas *of Porto Alegre, and approved by them. Prior to data collection, the women who agreed to participate in the study were given and signed an informed consent form containing all the information on the study. Regarding those under the age of 18, the informed consent form was signed by their legal guardians. The women were informed that they could decline to participate in the study. They were also informed that they could quit the study at any time, even if they had agreed to participate at first, and that their decision would not cause them any trouble.

### Study variables

Women were considered depressed when scoring 13 points or higher in the EPDS [17], validated for use [17,18] in Brazil. The EPDS [26] is a 10-item questionnaire that assesses the presence and intensity of depressive symptoms over the previous seven days, used with women as of the 6^th ^week after delivery.

The variables studies were grouped into characteristics:

- Demographic: age (reported in years), race/skin color (investigated by observation [27,28] and listed as white, black, yellow, native, mixed, and later regrouped as Caucasian and non-Caucasian)

- Socioeconomic: participant's years of schooling (the number of years completed at school), primary income earner's schooling (the primary income earner is defined as the person with the largest income in the household and the definition of schooling is the same as above), total income (taken as the total income of all people working and residing in the household in the month preceding the interview), per capita income (calculated by dividing the total income by the number of residents in the household and categorized in tertiles), employment/work (defined as any income-producing activity), marital status (defined by the presence of a partner or spouse).

- Religion: (the belief in or practice of a faith, cult or sect).

### Study implementation

The participants were selected from the Record of Living Newborn Infants of the State Health Department via software SPSS using a process based on pseudo-random numbers which choose a random sample from all records. Once the addresses were identified, the women were visited at their place of residence (home, hotel, boarding house and prison), with the interviews taking place between the 6^th ^and the 8^th ^week after delivery.

The data collection was carried out by 19 medical, psychology and social work students and professionals between the 7^th ^of July and the 30^th ^of August 2001. The interviewers were selected and trained by the main researcher, who also provided them with a booklet with instructions for questionnaire application and filling-out, including instructions on coding techniques.

The study was advertised in mass communications media 30 days prior to the beginning of data collection, in an effort to ease the access of interviewers to the selected women.

To conduct the interviews, it was established that 3 visits would be paid to each mother. In case interviewers were unsuccessful in their contacts with the mothers at the first 2 visits, the last visit would be paid by the head researcher. All the women contacted were given oral and written information on the research, signed the free and informed consent form and answered the questionnaire.

At the end of the interview, women presenting a positive score in the EPDS were recommended to seek outpatient treatment at the *Hospital de Clínicas de Porto Alegre*.

A special outpatient service was set up to treat women suspected of suffering from postnatal depression. The women who failed to contact the service were called upon and urged to see the specialists.

Besides the women mentioned above, women scoring 10 or higher in the scale were also invited to seek the outpatient service [26], according to the author's instructions.

In order to ensure the quality of the information collected, the interviewers received training in the application of the questionnaire, the guidelines in the instruction booklet were standardized and their fieldwork was supervised. Five percent of the randomly selected households were visited again by the research coordinator for the sake of quality control. All data entries used a duplicate field to eliminate errors.

### Data analysis

The association between the risk factors and PND was investigated through bivariate analysis using Pearson's chi-square test, and Cox's regression was used to calculate the prevalence ratios with time to the outcome assumed as constant [29,30], using statistical package (STATA release 7.0). Student's t-test was used to analyze the continuous variables.

**Figure 1:**
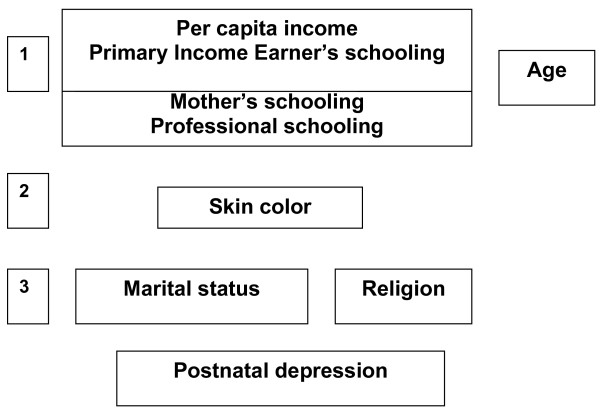
Hierarchical model for the analysis of demographic, socioeconomic and cultural risk factors for PND.

In order to identify independent risk factors, multivariate analysis was performed using hierarchical levels with a predefined model (Figure 1) that took into account the time relationship between PND and the risk factors. The risk factors investigated were per capita income, primary income earner's schooling, age, participant's schooling, employment, skin color (investigate by observations with proxy of ethnicity. That is, it include aspects sociocultural that exceeded the socioeconomic level) and marital status.

The model prioritized economic determinants over social characteristics and the chronology of their relationships in the hierarchization of the studied variables. The variables nearer the top of the figure were assumed to influence all other factors directly or indirectly [31], with the exception of age.

The determinants were selected in each level according to their statistical significance (value P < 0.2) to avoid [32] excluding a potential confounding factor. The variables with statistical significance P < 0.05 were retained in the model.

According to the model (Figure 1), the variables were analyzed and adjusted hierarchically in relation to those located on a level immediately above them. The variables associated with PND were retained in the model after adjusting for the confounding variables on the same level and for those hierarchically above.

In the first stage, the effect of the variables at the top hierarchical level was analyzed (effect of per capita income and primary income earner's schooling on PND) with no adjustment for the variables at the bottom hierarchical levels. In the second stage, the model retained the variables that were statistically significant in the first stage, and the participant's schooling, employment and skin color were added. These were inter-adjusted and were also adjusted for the statistically significant variables of the hierarchical level immediately above. Finally, marital status was added to the analysis after adjusting for the variables that had remained in the model in the previous stages.

## Results

Of the studied population, 300 women were selected, of which 271 women (90.3%) were recruited, there being three refusals and 26 failures to locate (18 women who did not reside in the addresses supplied and eight addresses which were not located). In average, the mothers were interviewed 7.1 (± 1.8 SD) weeks after delivery.

The prevalence rate of PND assessed through an EPDS score of 13 or above was 20.7% (95% CI 15.7–25.7). Of the total of 94 (56 with an EPDS score of ≥ 13 + 38 women scoring ≥ 10), 11 women sought treatment at the *Hospital de Clínicas de Porto Alegre *from July to December/2001.

The characteristics described in Table 1 show that approximately half of the women were 20 to 29 years old, 64% were Caucasian, 61% did not have employment/work, 83% lived with a spouse or partner, 67% had a monthly per capita income of up to 1.16 times the local minimum wage (monthly minimum wage = US$ 204, July 2007) and 51% had no religion. On average, these women had completed 8.35 years at school.

**Table 1:** Association between demographic and socioeconomic risk factors and PND, Porto Alegre, 2006.

		**Edinburgh ≥ 13**	**Crude Prevalence Ratio (95% CI)**	**Adjusted Prevalence Ratio (95% CI)**
				
	**n**	**% or mean ± SD**		
**Age (years)**				
14 – 19	43	23.3	1.30 (0.68 – 2.49)	
20 – 29	134	17.9	1.00	
30 – 39	17	22.7	1.27 (0.73 – 2.20)	
40 – 47	19	26.3	1.47 (0.64 – 3.39)	
P value			0.7	
				
Per capita income (MW)*++				
1.17 – 17.0	85	7.1	1.00	1.00
0.51 – 1.16	84	20.2	2.87 (1.19 – 6.92)	3.07 (1.09–8.69)
≤ 0.5	87	29.9	4.25 (1.84 – 9.77)	4.40 (1.48–13.0)
P value			< 0.01	0.03
				
Primary Income Earner's schooling**++	254	7.90 (± 6.02)+	0.96 (0.92 – 0.99) 0.04	1.01 (0.97–1.06) 0.7
P value				
				
Caucasian**				
Yes	175	16.6	1.00	1.00
No	96	28.1	1.70 (1.07 – 2.69)	0.80 (0.49–1.32)
P value			0.04	0.4
				
Employment/work**				
Yes	105	15.2	1.00	1.00
No	166	24.1	1.58 (0.93 – 2.68)	1.05 (0.61–1.79)
P value			0.1	0.9
				
Participant's schooling**	271	6.48 (± 3.55)+	0.90 (0.86 – 0.96)	0.98 (0.90–1.06)
P value			< 0.01	0.6
				
Spouse/partner**				
Yes	224	17.4	1.00	1
No	47	36.2	2.08 (1.29 – 3.34)	1.61 (0.97–2.66)
P value			< 0.01	0.06
				
Religion/Faith**				
Yes	132	18.9	1.00	1.00
No	139	22.3	0.85 (0.53 – 1.36)	0.78 (0.39–1.05)
P value			0.6	0.3

*Mw: (US$ 204, July 2007)

+ Mean and standard deviation

** Prevalence ratio adjusted for per capita income

+ Mean adjusted for per capita income

++ Losses due to refusal to respond or storage failures.

Table 1 shows that age was not associated with PND; however, non-Caucasian skin color, lack of spouse or partner, lower participant's and primary income earner's schooling and lower per capita income were significantly associated with PND in the bivariate analysis. The effect of income became greater after adjusting for confounding factors. In the non-adjusted analysis, women who did not live with their husbands or partners had twice the risk of developing PND when compared with women who lived with a spouse. After adjusting for per capita income, this variable showed a trend towards significance.

In the bivariate analysis, non-Caucasian women showed a prevalence of PND nearly two times higher than that of Caucasian women. However, after controlling the confounding factors, the effect of ethnicity was reduced considerably. Similarly, the participant's and the primary income earner's schooling acted as protective factors in the bivariate analysis, with every additional year of schooling by the mother translating into a 10% increase in protection against PND. After adjustment, these variables lost their significance.

## Discussion

This population-based study was carried out in a random sample of mothers living in a large city of a developing country, whose children were born in June, 2001, and had a non-recruitment rate of less than 10%. These mothers are thought to be similar to those giving birth in other months of the year.

The cross-sectional design employed makes it difficult to conduct a temporal evaluation between causes and possible effects once that the factors under study and the clinical outcome are studied simultaneously.

Another potential limitation resides in the size of the sample, which may have not been adequate to allow the detection of an association between the determining factors and the outcome.

The study detected that 20.7% of 14 to 47 year-old puerperal women in Porto Alegre suffered from PND. This prevalence rate is considerably higher than that described in Rio de Janeiro [17], where on the third postnatal month, 12% of low-income women, identified by the Society of Dwellers of the District of Anaia and invited to participate in the study, suffered from PND. In Brasilia [18], a PND prevalence rate of 13.4% was found by a study of 236 middle-class women between the 6^th ^and the 24^th ^postnatal week, identified from the record of live births, located through the phone directory and invited to participate in the study. These studies used the EPDS with different cutoff points, 13, in Rio de Janeiro, and 12, in Brasilia.

Differences in methodology such as study population, sample size and sampling selection, diagnostic criteria and postnatal period elapsed can account for disparity in prevalence rates.

The rate of PND in Porto Alegre is higher than the 12% rate found in a meta-analysis [24] of studies using the EPDS (cutoff equal to or greater than 12), with 3,121 women residing in industrialized cities in Western countries. It is similar to the rate of 23% detected in Goa, India [21], in a consecutive sample of 270 women from a prenatal clinic, where the EPDS was assessed (score ≥ 12) between the 6^th ^and 8^th ^postnatal week. The prevalence of PND found in developing countries is usually greater than rates established in industrialized countries, giving rise to questions concerning which other factors could be affecting these results, such as the socioeconomic level.

Regarding those women who were contacted once again and urged to seek assistance, we identified the following reasons as the main obstacles mentioned to justify failure in keeping up appointments or non-adherence to treatment at the *Hospital de Clínicas de Porto Alegre *outpatient service: difficulty in grasping the seriousness of the condition presented, distance of up to 50 km from the service location, financial inability to afford the commute and lack of a sitter to look after the newborn and other small children.

Unlike other studies [15,20], our study did not find an association between age and puerperal depression. Studies investigating the association between age and PND have shown conflicting results. There are studies [15] showing that women over 29 are at increased PND risk. In contrast, another study [20] shows that older age was considered a protecting factor against the onset of puerperal depression.

The inconsistent associations with socioeconomic and religion-related determinants may be due to the lack or insufficient adjustment for confounding factors. Socioeconomic variables are frequently modeled in statistical analyses without due concern for the pathways through which they act or the potential for interactions among income, education and the other variables. In this study, socioeconomic variables such as mother's and primary income earner's school education, Caucasian skin color, and presence of spouse or partner presented a statistically significant association with PND only in the bivariate analysis.

Per capita income remained independently associated with PND, thus confirming the findings [16,21,24] in the literature. However, most of the associations were no longer significant after the adjustment for income. An explanation for this finding is the effect of per capita income on the mediation of other socioeconomic risk factors. If the primary income earner's schooling is stratified into per capita income levels, we can observe that among those individuals with low school attendance only those with higher per capita incomes are able to notice the signs of postnatal depression. In addition, the effect of education may be due to the varying influence of sociocultural factors, such as the degree of acculturation or modernization.

Religious beliefs showed no significant association in the present study, a result that contradicted our expectations given that Brazil is seen as a very religious country under great influence of Christian faiths. Our hypothesis, which was in agreement with findings in the literature [33], was that having some sort of religious faith would act as a protecting factor against puerperal depression.

## Conclusion

The PND prevalence found is high, pointing towards the need to consider it an issue of public health given the already identified adverse effects on the mother, the newborn and the family while the condition remains untreated, requiring public policies for its prevention and treatment.

Of the variables studied, only income remained as an associated factor to PND after the adjustment. The hierarchical analysis allowed us to detect that most of the effect of education and religion was explained by income, but having a partner or spouse remained nearly significant toward protection.

In low income populations, women should be routinely evaluated for postnatal depression, and those with no partner or spouse are likely to require further care from health services and should be given the benefit of health prevention programs.

## Abbreviations

PND: Postnatal depression.

## Competing interests

The author(s) declare that they have no competing interests.

## Authors' contributions

LT: conceived of the study and its design, coordinated the acquisition of data, made substantial contributions to data analysis and interpretation, and drafted the manuscript.

LPG: contributed to the conception and design of the study, and carried out data analysis and interpretation.

SCF: carried out the critical revision for important intellectual content.

ED'AB: contributed to the conception of the study, helped to analyze and interpret the data and gave final approval of the version to be published.

All authors read and approved the final manuscript.

## Pre-publication history

The pre-publication history for this paper can be accessed here:


